# General Practitioners’ Attitudes Toward Artificial Intelligence–Enabled Systems: Interview Study

**DOI:** 10.2196/28916

**Published:** 2022-01-27

**Authors:** Christoph Buck, Eileen Doctor, Jasmin Hennrich, Jan Jöhnk, Torsten Eymann

**Affiliations:** 1 Department of Business & Information Systems Engineering University of Bayreuth Bayreuth Germany; 2 Centre for Future Enterprise Queensland University of Technology Brisbane Australia; 3 Project Group Business & Information Systems Engineering Fraunhofer Institute for Applied Information Technology Bayreuth Germany; 4 Finance & Information Management Research Center Bayreuth Germany

**Keywords:** artificial intelligence, AI, attitude, primary care, general practitioner, GP, qualitative interview, diagnosis, clinical decision support system

## Abstract

**Background:**

General practitioners (GPs) care for a large number of patients with various diseases in very short timeframes under high uncertainty. Thus, systems enabled by artificial intelligence (AI) are promising and time-saving solutions that may increase the quality of care.

**Objective:**

This study aims to understand GPs’ attitudes toward AI-enabled systems in medical diagnosis.

**Methods:**

We interviewed 18 GPs from Germany between March 2020 and May 2020 to identify determinants of GPs’ attitudes toward AI-based systems in diagnosis. By analyzing the interview transcripts, we identified 307 open codes, which we then further structured to derive relevant attitude determinants.

**Results:**

We merged the open codes into 21 concepts and finally into five categories: concerns, expectations, environmental influences, individual characteristics, and minimum requirements of AI-enabled systems. Concerns included all doubts and fears of the participants regarding AI-enabled systems. Expectations reflected GPs’ thoughts and beliefs about expected benefits and limitations of AI-enabled systems in terms of GP care. Environmental influences included influences resulting from an evolving working environment, key stakeholders’ perspectives and opinions, the available information technology hardware and software resources, and the media environment. Individual characteristics were determinants that describe a physician as a person, including character traits, demographic characteristics, and knowledge. In addition, the interviews also revealed the minimum requirements of AI-enabled systems, which were preconditions that must be met for GPs to contemplate using AI-enabled systems. Moreover, we identified relationships among these categories, which we conflate in our proposed model.

**Conclusions:**

This study provides a thorough understanding of the perspective of future users of AI-enabled systems in primary care and lays the foundation for successful market penetration. We contribute to the research stream of analyzing and designing AI-enabled systems and the literature on attitudes toward technology and practice by fostering the understanding of GPs and their attitudes toward such systems. Our findings provide relevant information to technology developers, policymakers, and stakeholder institutions of GP care.

## Introduction

### Overview

As artificial intelligence (AI) enabled systems have surpassed human performance in different aspects of economy and society, the increasing technological maturity and widespread applicability of such systems is leading to skyrocketing expectations [1]. The technological progress in various fields such as machine learning, robotics, big data analytics, decision support systems (DSSs) as well as the ubiquity and availability of data and the prevalence of information systems (ISs) are opening previously unavailable value creation potentials [2-5]. We understand AI as a set of value-adding technological solutions that use self-learning algorithms to perform cognitive tasks at a level comparable with that of humans [6]. Various AI solutions that provide decision support typically associated with human cognition are emerging and hold the potential to reshape the nature of work [1,7-9]. Thus, AI is also a promising approach for the health care domain [10]. AI and related technologies, such as big data analytics and DSSs, are distinct phenomena with important conceptual differences; however, some of the underlying technologies might overlap. In health care, AI technology advances health information technologies such as clinical DSSs (CDSSs). These systems assist medical professionals in tasks related to medical decision-making [11], such as diagnosis, prescription, or the prevention of medication errors [12,13]. Among others, typical functions are alerts, reminders, and recommendations [14,15].

There are two forms of CDSSs in health care: knowledge-based systems and non–knowledge-based systems. Knowledge-based systems match their knowledge base with individual patient characteristics and make decisions based on preformulated rules [16,17]. As such, knowledge-based CDSSs are designed to inform skilled actors. That is, to provide actors in the health care system, for example, physicians, with relevant information to comprehend internal and external structures and processes. On the other hand, non–knowledge-based CDSSs use AI technologies, which eliminate the writing of rules and the need to follow expert medical input. This integration of AI technology allows the CDSSs to learn from experience and find patterns in medical data [18]. Hence, the vision of AI is to enable systems to be on human-level intelligence. Here, intelligence refers to an agent’s ability to achieve goals in a wide range of environments [19,20] and goes beyond the mere preparation of information. Instead, AI highlights the ambition to develop artificial agents that are able to learn, decide, and act autonomously [9,21].

AI-enabled systems have already successfully entered various subdisciplines of health care, such as image recognition, diagnosis, and precision medicine [10]. Most AI-enabled systems have immediate relevance in health care and several potentials for value creation, such as higher efficiency and accuracy in diagnosis and lower error rates [22-24]. Furthermore, AI-enabled systems are more enduring in repetitive tasks than humans, thus enhancing cost-efficiency [25].

Regarding these promised benefits, AI-enabled systems have particular potential in the field of primary care. General practitioners (GPs) serve as the first point of medical contact and therefore must diagnose with high levels of uncertainty and under high time pressure. For instance, in Germany, primary care is one of the most frequently used health care services, leading to an average physician–patient contact time of 7.6 minutes [26]. Moreover, GPs are responsible for the initial diagnosis, thus setting the direction for whether a patient receives the right care. Misdiagnosis in this early stage of diseases can have severe impact on medical quality in terms of injuries, avoidable illnesses, hospitalizations, and in 10% of cases, death [27,28]. Besides the potentially tragic individual consequences, such misdiagnoses also increase the cost of care [29].

To prevent these risks, the health care system depends on innovative, reliant, and fast approaches to decision-making processes in GP care [30]. Considered as an integrative system, AI-enabled systems free up physicians’ time for more sophisticated tasks [31]. Furthermore, AI-enabled systems can ensure stronger physician–patient relationships [32], which is especially valuable in GP care as it enables the therapeutic benefit of improved continuity of care and more holistic and individualized treatments [32]. In addition, AI-enabled systems can reduce diagnostic errors, which are considered the greatest threat to patient safety in GP care [33].

Although AI-enabled systems in primary care diagnosis are gradually becoming feasible and useful, their widespread implementation still remains a future scenario [10,34]. Among others, reasons for the slowdown in adoption are the physicians’ lack of trust in [35,36] and acceptance of [16,37] AI-enabled systems. These adoption barriers arise, for instance, from the concern that AI-enabled systems might be trained with a heterogeneous database owing to the diversity and individuality of medicine, leading to biased or overadapted outcomes. Overcoming these hurdles requires balancing the GP’s trust in AI-enabled systems [35]. On one hand, developing trust in such a system is beneficial to its adoption and use. On the other hand, AI-enabled systems may bear risks when physicians blindly rely on such systems’ suggestions and outcomes. Furthermore, factors such as the anticipated threat to professional autonomy and legal liabilities from using AI-enabled systems are hindering factors, as known so far [38].

A key driver for successful implementation and uptake of AI-enabled systems is the attitude of physicians. By discussing our findings on GPs’ attitudes toward AI-enabled systems within a facilitating context for practical implementation, we extend the previous work of Blease et al [39], who recognized the relevance of the topic and investigated the opinions of GPs about the possible impact of AI on GP care.

### The Construct of Attitude as Our Theoretical Lens

We understand attitude as a psychological tendency that determines how GPs evaluate their favor or disfavor against AI-enabled systems [40]. Following Rosenberg and Hovland [41], the most widespread construct of attitude—the 3-component model—comprises the affective, cognitive, and behavioral dimensions of attitude. First, the affective component refers to the respondent’s emotional reaction to an attitude object, including their empathy, preferences, and feelings. Second, a person’s thoughts and beliefs toward an attitude object form the cognitive component, which includes the individual’s idea, opinion, or knowledge of it [41]. Third, the behavioral component rests on the attitude behavioral consistency assumption, described as the extent to which an attitude predicts a behavior, including the willingness or intention to act to deal with an object [41,42]. Overall, the attitude construct assumes a consistent and dependent relationship among the affective, cognitive, and behavioral components, suggesting that a change in one component leads to changes in the other components [41].

**Figure 1:**
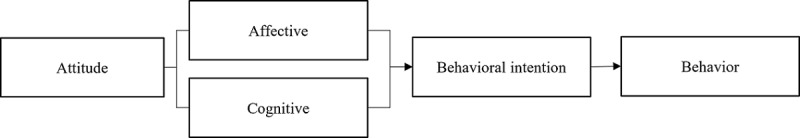
The 2-component model of attitude [44,45].

However, researchers acknowledge that behavioral intention (ie, the behavioral component) does not always correspond to the feelings (ie, the affective component) and opinions (ie, the cognitive component) [42]. This challenges the behavioral component as an integral part of the attitude construct. Thus, the 2-component model of attitude was developed based on this critique (Figure 1) [43]. According to this model, attitude consists of an affective and a cognitive component that simultaneously form the behavioral intention, which—in turn—explains a de facto behavior [44].

Extant work describes behavioral intention as the mediator in the relationship between attitude and behavior [46,47]. Thus, it is assumed that the stronger the intention, the higher the likelihood of the behavior occurring [48]. According to the principle of compatibility, behavior is only predicted by attitude to the extent of both being on the same level of specificity or generality regarding their objective, context, and time elements [49]. However, regardless of the intensity of influence, there is broad agreement that attitude fosters behavioral intention [50]. Drawing on its relevance to users’ subsequent intentions and behavior, we use the attitude construct to foster our understanding of how to better exploit AI-enabled systems’ potential in GP care and promote their future use.

Different quantitative studies have investigated the relationship between attitude and the intention to use AI-enabled systems, for example, regarding medical students [51,52]. In this context, research theories such as the unified theory of acceptance and use of technology (UTAUT) and the theory of reasoned action find application. These established approaches of technology acceptance research, originating in social psychology, primarily focus on users’ intentions [48,53-55]. However, these approaches do not provide a comprehensive understanding of GPs’ attitudes toward AI-enabled systems, as they use abstract constructs and variables and do not capture detailed, even emotionally based, and spontaneous responses from the potential users [56]. However, apart from this, there is a more important criterion why we chose not to use these models for our study. GPs rarely use AI-enabled systems so far [34], so research in this respect considers a rather hypothetical use scenario than actual use. Investigating the intention to use, which is a direct determinant of the actual use according to UTAUT, is therefore not feasible as the possible system features and functions are not yet available. Nevertheless, it is possible to look at the underlying attitude toward the technology, which exists outside the de facto experience of use.

### Goal of This Study

Despite the relevance of AI technologies in the health care sector, a profound understanding of GPs’ attitudes toward AI-enabled systems and their underlying determinants is still lacking. Therefore, the purpose of this study is to investigate which determinants influence GPs’ attitudes toward AI-enabled systems in diagnosis. We see this as an important step in developing user-centered solutions, which will positively affect the intention to use and support the successful introduction of AI-enabled systems in primary care.

## Methods

### Data Collection

To identify the determinants of GPs’ attitudes toward AI-enabled systems in diagnosis, in-depth insights are vital. Following the interpretative paradigm, qualitative methods were used to obtain an understanding of individuals’ technological attitudes in the medical context [57]. Thus, we did not prescribe and narrow the phenomenon to only the testing of variables but emphasized the complexity of human understanding and behavior [58].

Data collection followed an interplay of continuous and iterative matching steps of sample selection (recruiting of participants), interview guideline creation (and improvement), data collection (interview conduction), data analysis (transcription and coding), and revision of the process steps. As the iterative process and constant comparison make it challenging to provide a timeline or sequence of these steps, it is reflected upon as a constant effort in creating a comprehensive and growing understanding of the participants’ attitudes, which are not always distinctively observable [59,60]. In terms of saturation approaches, this study emphasized the term *conceptual depth* proposed by Nelson [61], whereby researchers cumulatively judge the sufficiency of depth of understanding, thus allowing for incremental development. Following Schultze and Avital [60], the choice of semistructured expert interviews allowed focusing on the research topic while also providing in-depth information [62]. This approach offered a modular structure through which the participants could access and reflect upon their experiences and perceptions regarding AI-enabled systems. We derived overarching interview topics from the given practical research objective and through reflective discussions within the author team, resulting in the exploratory interview questions. The 4-phase process to interview protocol refinement, proposed by Castillo-Montoya [63], served as a basis for developing the interview guideline, including pretesting the first version of the interview guideline with three volunteers: a health economist, a nurse, and a physician. The first version of the interview guide addressed the topics of personal experiences, assessments of perceived diagnostic support, design requirements, and motivations for the use of AI-enabled systems in a broader perspective. It underwent 9 iterations, receiving more detailed and tailored questions about the research topic with each interview. The final interview guideline (Multimedia Appendix 1) was designed to question the participants on their understanding of AI-enabled systems and provide this paper’s literature-based definition, aiming toward a shared understanding of AI-enabled systems and comparable interview results. Owing to the nature of the semistructured interviews, the results were not limited to collecting attitude determinants. In addition, this approach allowed us to capture insights into GP care’s challenges and special characteristics, which contributed to a profound understanding of the determinants of attitudes in the medical context.

The interviews took place both in person and via phone and anonymity was guaranteed to all participants within the study. As face-to-face interviews create a trusting and comfortable atmosphere and enable more detailed information on participants’ feelings and attitudes, the interviewers preferred them for data collection [64]. With the participants’ consent, audio recording and transcription was performed to allow thorough data analysis by using a software program for qualitative and mixed methods data analysis, named *MAXQDA 2020* (VERBI GmbH).

### Data Analysis

For analyzing the interview transcripts, grounded theory analysis techniques were applied. As stated by Glaser [65], the traditional grounded theory methodology (GTM) seeks to develop a conceptual theory that depicts a relevant or problematic behavior pattern (here, GPs’ attitudes toward AI-enabled systems in diagnosis). GTM focuses on behavioral aspects where attitude behaves as an antecedent and is therefore equally suitable for application. Applying the GTM approach allowed to handle the unstructured qualitative data sets, discover relevant categories and relationships among them, and contextualize and interpret them [66]. According to GTM, the analysis begins with the first collected data set, as the experiences with the first interview process already influence the researcher and thus the upcoming interviews. In the interview process, participants’ responses and clarified check-backs were closely scrutinized and documented [67]. This knowledge about misconceptions was considered in the iterative development of the interview guidelines. Furthermore, it allowed the clarification and precise alignment of the research question [66].

The interview data were paraphrased into relevant bits (open coding) in line with the 3-step Straussian approach for coding (open, axial, and selective coding). Thus, the first step consisted of an initial and careful reading of the interview transcripts, highlighting any phrases that may have proven to be relevant to the research topic. Over the course of data analysis, 307 open codes emerged. Following the Glaser and Strauss [68] specifications, the codes were further examined and paraphrased, merging those with common themes into concepts. Thus, we assigned special value to the wording of and syntactical differentiation among expressions. After comparing the allocation of the concepts, they were merged into categories. Moreover, the relationships among them were identified, which refers to the axial coding step. By setting all elements in relation to one another, the core category *attitude determinants* was distinguished from other categories (selective coding) [66]. In line with GTM, the 3 coding steps followed a flexible and iterative process instead of a fixed sequence [68].

For enhanced validity of the coding results, 2 authors (JH and ED) performed card-sorting allocation. Thus, the open codes and concepts identified by 1 author (JH) in the first round served as the foundation for the second author (ED) but in an unmatched format. The second author conducted a blind card-sorting round with this groundwork and commented on the constructs and documented challenges that arose in the allocation of open codes to a specific construct. This second author further added open codes that were not initially identified during the process. In case of deviations in matching open code to constructs between the 2 authors, the entire research team discussed the said allocations. An agreement was found in all cases of card-sorting deviations. Furthermore, in all coding rounds, the authors iteratively discussed the constructs’ abstraction levels and their various definitions and revisited their coding results for adjustments, which the literature refers to as *constant comparison method* [66]. Whenever the authors gained new insights from their constant comparison and iterations, they repeated the open coding steps for all the interview sets backward and forward.

## Results

### Descriptive Results and Study Population

We interviewed 18 GPs from Germany between March 2020 and May 2020, selecting them via convenience sampling [69]. Thereby, we contacted 110 physicians within the geographic reach of the research team via mail and further relied on personal network contacts. In addition, we asked the acquired participants for the contact information of other colleagues who might be interested in participation. All participants had at least 1 year of work experience in GP care [69]. Of the 18 GPs, 7 (39%) were situated in urban areas with a range of 75,000-127,000 inhabitants, whereas 11 (61%) participants were situated in rural and small-town areas with a range of 3200-23,000 inhabitants. For a more accurate evaluation of the participants’ statements in light of relevant demographic and structural data, individual characteristics of the participating GPs and descriptive characteristics of our data collection are shown in Table 1. We further report the specifics of the interview lengths and styles.

**Table 1:** Descriptive characteristics of the participants and the data collection (N=18).

Participant number	Age (years)^a^	Gender	Working situation	Interview duration (minutes)^b^	Interview style
GP^c^ 1	70	Female	JP^d^	27	In person
GP 2	51	Male	JP	28	In person
GP 3	50	Male	JP	31	Via phone
GP 4	41	Male	JP	22	In person
GP 5	52	Female	JP	36	In person
GP 6	50	Female	—^e^	—	—
GP 7	50	Female	JP	23	Via phone
GP 8	36	Female	JP	25	In person
GP 9	45	Female	JP	23	Via phone
GP 10	58	Male	JP	46	In person
GP 11	38	Male	IP^f^	30	Via phone
GP 12	44	Female	JP	35	Via phone
GP 13	52	Male	JP	60	Via phone
GP 14	43	Female	JP	25	Via phone
GP 15	40	Male	GPC^g^	29	Via phone
GP 16	34	Female	IP	40	In person
GP 17	47	Male	JP	23	Via phone
GP 18	51	Male	IP	44	Via phone

^a^Mean age is 47.33 (SD 8.31) years.

^b^Mean interview duration is 30.38 (SD 10.06) minutes.

^c^GP: general practitioner.

^d^JP: joint practice.

^e^GP 5 and GP 6 participated in the interview together.

^f^IP: individual practice.

^g^GPC: general practitioner center.

### Three-Step Coding Results

#### Overview

We describe the 5 categories and 21 concepts that determined our GPs’ attitudes toward AI-enabled systems as derived from our qualitative data sets. Our baseline for considering the attitude determinants was the AI literacy level among the participants. Long and Magerko [70] defined AI literacy “as a set of competencies that enables individuals to critically evaluate AI technologies; communicate and collaborate effectively with AI; and use AI as a tool on the web, at home, and in the workplace.” Hence, the identified attitude determinants depend on the participants’ statements and their knowledge regarding AI-enabled systems, irrespective of whether this knowledge is true to facts. Most participants had poor AI literacy in the data set and had not yet interacted with AI-enabled systems. For example, the self-learning ability of AI-enabled systems was known to only 33% (6/18) of the respondents. Although these 6 GPs were familiar with this AI technology component, they often did not fully understand what AI is. For example, GP 3 mentioned the following:

In the end, every time I turn on a computer, I use artificial intelligence.

Participant 3

Only 22% (4/18) of the GPs had experience with AI-enabled systems and only 50% (2/4) of them explicitly mentioned having used it in their GP work. In answering the question of why GPs had not had experiences with AI-enabled systems, the participants gave 3 explanations. First, they said they did not know about any AI-enabled tools for the GP sector (interview 15). Second, they did not see the necessity to use AI-enabled systems (interview 9). Third, is a general aversion toward the use of technology in medicine (interview 8). Although most participants had not had contact with AI-enabled systems, most GPs agreed on the role of the AI-enabled system in GP care in the future. A participant said the following:

You cannot decide against [AI technology] because it will come. Because without [AI technology] [diagnosis] is not possible.

Participant 1

The participants associated expected time effort with the use of AI-enabled systems in routine diagnoses owing to the necessary AI technology integration into an established and effortless routine process. Therefore, the participants limited the scope of its application to cases of rare diseases and to cases in which the physicians could not reach a diagnosis without additional help (interview 8).

When grouping the statements, we paid particular attention to the wording and syntactic differentiation that the physicians used in their answers. The interview data revealed 5 main categories that summarize the influencing determinants of GPs’ attitudes toward AI-enabled systems in diagnosis. When we raised questions on potentially using AI-enabled systems in clinical practice, the GPs had various *concerns* and *expectations*. In addition, we found that the *environmental influences* and certain *individual characteristics* influenced their attitudes. Whenever GPs stated that AI-enabled systems must meet certain requirements for them to consider using it, we categorized them as *minimum requirements of AI-enabled systems*. Table 2 shows an overview of all the categories and concepts, which is followed by a description of the determinants, as supported by interview quotes.

**Table 2:** Overview of the categories and concepts.

Determinants of attitudes toward AI^a^-enabled systems and concepts			Open codes in each concept		Open codes in each category	
**Concerns**						57
	Existential anxiety	12				
	Change of the physician–patient relationship	7				
	Misuse of data	14				
	Diagnostic bias	24				
**Expectations**						112
	Diagnostic quality	35				
	Diagnostic efficiency	19				
	Legal liability	4				
	Lack of human competencies	43				
	Time expenditure	11				
**Environmental influences**						37
	Changing working conditions	8				
	Stakeholder influences	13				
	Media	12				
	Information technology infrastructure	4				
**Individual characteristics**						17
	Age	11				
	Affinity with technology	6				
**Minimum requirements of AI-enabled systems**						84
	Time efficiency	40				
	Diagnostic quality	15				
	Data security	10				
	Economic viability	12				
	Transparency	3				
	Autonomy	4				

^a^AI: artificial intelligence.

#### Concerns

##### Overview

*Concerns* include all doubts and fears concerning AI-enabled systems. Overall, this category consists of four concepts: (1) existential anxiety, (2) change of the physician–patient relationship, (3) misuse of data, and (4) diagnostic bias.

##### Existential Anxiety

Half (9/18, 50%) of the participants expressed *existential anxiety* connected with AI-enabled systems as they perceive that this technology can take over some of their tasks. GP 2 said the following:

At one point, the own decision and the own expertise threatens to be pushed into the background or to become redundant.

Participant 2

GP 14 also perceived the threat of being replaceable by AI-enabled systems and provided an example of an AI-enabled system that has achieved higher diagnostic accuracy than physicians. This concept included the fear of no longer being useful and being replaceable by AI-enabled systems and the worry of losing their unique status as physicians. A participant said the following:

Surely, many doctors probably see their unique medical status endangered, that they are under the surveillance of others, that they think there is a bit of an attack on their own vanity.

Participant 12

##### Change of the Physician–Patient Relationship

The participants mentioned that AI-enabled systems could threaten the physician–patient relationship. Endangerment of this relationship, which fundamentally defines GP care, further compromises appropriate patient care (participant 3). As patients could feel that the AI-enabled system performs the treatment, the physicians assumed that the use of AI-enabled systems might negatively impact the physician–patient relationship (participant 11). In this regard, a participant mentioned the following:

Since [the patient] has the feeling [...] that the machine takes care of it and the doctor would only have to put his signature under it.

Participant 11

Participants mentioned the impairment of the physician–patient conversation through the use of technology as threatening to the physician–patient relationship. The concern is that, by using AI-enabled systems during patient consultations, a GP cannot devote all their attention to the patient sitting in front of them, but instead must also focus on the screen to follow an AI-enabled system’s recommendations. A participant commented the following:

[The treatment] may drift off into a standardized interview, and that’s probably not necessary.

Participant 12

GP 13 was concerned that AI-enabled systems would generally reduce physician–patient contact, which is a core component of GP care and is inevitable for successful treatment and patient care. The potential endangerment of the physician–patient relationship by the use of AI-enabled systems was also often linked to misuse of data.

##### Misuse of Data

With the use of AI-enabled systems and the disclosure of both the patients’ and physicians’ data, *misuse of data* is a key concern and impacts GPs’ attitudes toward AI-enabled systems. In this context, GP 3 saw the problem in the connection between AI-enabled systems used in practice and the interconnectedness between these systems and the internet:

[AI-enabled systems] are not stand-alone systems but are networked, and [...] actually, work over the internet with such simple things as voice recognition. And in my view, this will change the doctor-patient contact considerably. [...] I consider the fundamental trust in the patient-physician-conversation [...] to be a very important basis for our work. And I also see [the trusting relationship between the patient and the physician] as being in danger due to the increasing use of such procedures. I find this very worrying.

Participant 3

Internet access makes the data accessible and renders the patient and physician transparent, thus violating data privacy and leading to serious consequences for patients. A participant described it as follows:

Patient data are very sensitive data. Disease data are very sensitive data. [There is the risk that] they are passed on somewhere, that some authorities who have nothing to do with it or should have nothing to do with it could intercept the data and use this to the disadvantage of the patients.

Participant 11

Thus, the physicians are concerned about the data being misused by other stakeholders as supported by GP 4:

The problem is that large companies use AI to gain access to lucrative patients and to control them via AI.

Participant 4

Further, GP 3 warned of the danger of pharmaceutical companies programming AI-enabled systems for their purposes, referring to medication proposals that are not medically indicated but instead deliver a monetary benefit for the producing company. They justified this concern with experiences from working with other technologies (participant 3). This concept also summarizes physicians’ concerns about being monitorable and controllable at work when using AI-enabled systems. Owing to connection to the internet, GP 10 assumed that every step of physicians will be transparent and can be monitored. However, the GPs did not explicitly mention who would have interests in observing and controlling them.

##### Diagnostic Bias

According to the participants, AI-enabled systems can cause *diagnostic bias*, whereby the technology influences the GP’s decision-making in ways that can negatively affect the course and success of treatment. Once a GP has received suggestions from an AI-enabled system, they may not consider further possible diagnoses (participant 11). In this context, GP 8 spoke of the fear of being put on a completely wrong track and the likelihood that the AI-enabled system indicates a diagnosis that does not fit and therefore leads a GP to mistreat the patient. A frequent concern was that physician might become overreliant on the technology, neglecting their own medical and experience-based knowledge. Furthermore, the participants also mentioned the risk of overexpansion of treatment services as supported by participant 17:

The AI will recommend examinations that I would personally put last, ie. it will possibly lead to so-called device medicine, involving a lot of safeguard diagnostics, which I consider to be quite questionable.

Participant 17

#### Expectations

##### Overview

Besides *concerns*, we also found *expectations* to be determinants of GPs’ attitudes. This category reflects GPs’ thoughts and beliefs about AI-enabled systems’ expected benefits and limitations regarding GP care. Although the expected benefits had a positive connotation in the interview data (concepts regarding *diagnostic quality*, *diagnostic efficiency,* and *legal liability*), the expected limitations depicted a negative perspective (concepts encompassing statements relating to a *lack of human competencies* and *time expenditure*).

##### Diagnostic Quality

*Diagnostic quality* represents the expectation that AI-enabled systems can improve the quality of care via more accurate and precise diagnosis. It is GPs’ job to provide patients with the best possible care, which is why the expected benefits of AI-enabled systems positively influenced the GPs’ attitudes. Especially in rare diseases, which GPs do not regularly treat, the expectation from AI-enabled systems is an improvement of diagnostic quality as AI-enabled systems can work with a larger database than the human brain (participant 18). Thus, AI-enabled systems should act as support or a backup for the physician, in parallel or after a medical diagnosis. GP 12 assumed that AI-enabled systems could assist GPs in the decision-making process and thought that this would positively impact the outcome quality:

But for rarer diseases, when it comes to making a diagnosis; for example, a red skin spot that I can’t classify at all, then it would be conceivable [...] to reaffirm or reassure oneself [by means of AI].

Participant 12

Furthermore, the expectation from an AI-enabled system is that it is more enduring than humans. Unlike a physician, an AI-enabled system does not tire and its diagnostic quality does not suffer from human-like, lower-concentration performance during the course of a day. A participant said the following:

If AI is well programmed or if there are no failures in it, then AI is more accurate than a person, who is sometimes tired [and thus] makes bad decisions.

Participant 2

##### Diagnostic Efficiency

Besides the expected diagnostic quality, the participants stated that an AI-enabled system’s ability to make rapid diagnoses is a further expected benefit. We refer to this expectation as *diagnostic efficiency*. GP 2 transferred the time advantages of using AI-enabled systems to the area of image recognition and expected AI-enabled systems to be 3 times faster than a physician:

While a radiologist might manage 60 diagnostic findings a day, the AI could work day and night and deliver perhaps 180 or 200 findings. And if that happens with similar quality, then [...] you could examine many more patients than a human alone could.

Participant 2

On the basis of this benefit of AI-enabled systems, GP 14 expected the use of AI-enabled systems to influence disease progression positively. In addition, GP 1 emphasized the necessity of fast-working AI-enabled systems in the detection of health threats:

Now a completely new virus has appeared in China or Japan, and to get ahead of it, you need artificial intelligence which can detect [the virus] much faster.

Participant 1

*Diagnostic efficiency* included the GPs’ expectations regarding physician support via AI-enabled systems, reducing the daily workload by preselection (participant 7) and patient prioritization (participant 13). This time-saving effort would give GPs some relief and would allow them to concentrate on more serious cases (participant 7).

##### Legal Liability

*Legal liability* included the expectation that AI-enabled systems will give GPs legal backing. All decisions will be documented using AI-enabled systems, allowing the providers to prove the correct decision-making approach in a legal proceeding (participant 12). Furthermore, the participants added the assumption that AI-enabled systems could support the physician’s choice of treatment. In this context, GP 13 mentioned the following:

[With] AI, you can then understand how [the physician] came to a decision because AI said the risk was 0.001.

Participant 13

This was supported by the expectation of built-in legal protection and shifting responsibility from the GP *toward* the AI-enabled system (participant 4).

##### Lack of Human Competencies

Besides the above-mentioned positive determinants, the following *expectations* depicted the perceived limitations of AI-enabled systems. The expected *lack of human competencies* in AI-enabled systems was mentioned with a high emphasis. It included the GPs’ assumption that AI-enabled systems do not have certain human competencies, which are, in fact, crucial for adequate and appropriate treatment in GP care. The respondents agreed that AI-enabled systems will not—some said never—be able to have certain human competencies. In this context, empathy (participant 5), intuition (participant 1), gestures (participant 13), experience (participant 12), and clinical reasoning ability (participant 3) were mentioned. These competencies are important in GP care to collect all relevant information to be able to provide optimal care. Of the 18 participants, 2 (11%) participants said the following:

There is something behind almost every illness that makes [diagnosis] even more challenging. And if this is not considered, it will not be possible to help a patient comprehensively. And I think [AI] can probably not do this.

Participant 5

Experience can hardly be replaced by AI. Experience and intuition. And empathy. This is just how I treat people, to get something out of them. So, this is something that defines a good physician and cannot be replaced by AI. Empathy.

Participant 1

Furthermore, describing and verbalizing much of the information collected in GP care (such as mimics or gestures) is not always possible. However, it is an essential data input for the proper operation of any technology (participant 13). Participants expressed that many patients just make an appointment to have some human interaction, for instance, lonely older patients. GP 15 explained this as follows:

My experience every day with patients is that they want to be touched, and they want to look you in the eyes.

Participant 15

For them, AI-enabled systems seemed to be unable to fulfill these needs. In the context of human competencies, GP 13 underlined AI-enabled systems’ limitations:

People are certainly beaten by [AI] in many ways. But not in the emotional one.

Participant 13

##### Time Expenditure

*Time expenditure* included the expectation that in most cases, GPs would need more time for the *decision-making* process by involving AI-enabled systems, because in routine cases, GPs usually diagnose on their own within seconds. In this context, participant 11 commented the following:

[...] in routine cases, [AI] would not be a time saver for me.

Participant 11

With AI-enabled systems, additional effort is expected by the participants because they fear that data must be entered in the documentation and fed into the AI-enabled system. GP 2 assumed additional time expenditure owing to a person’s need to critically reflect on the results of the AI-enabled system.

#### Environmental Influences

Besides the 2 main categories, we also identified *environmental influences* that influence GPs’ attitudes toward AI-enabled systems. The summarized determinants include influences resulting from an evolving working environment (*changing working conditions*), the perspectives and opinions of key stakeholders (*stakeholder influences*), the available IT hardware and software resources (*IT infrastructure*), and the media environment (*media*).

##### Changing Working Conditions

*Changing working conditions* included GPs’ perspectives on the challenges caused by demographic change (participant 10), a changing spectrum of diseases (participant 1), and the constant increase in medical knowledge (participant 3). Regarding demographic change, GP 1 stated the following:

The lack of physicians comes with giant steps, and what is also urgently needed is telemedicine. And this, of course, needs AI with it.

Participant 1

However, demographic change also included the necessity to modernize a practice’s equipment with new technologies to be interesting for younger physicians (participant 10). AI-enabled systems were also considered necessary to stay updated about the increasing medical knowledge and provide the patients with the best and latest information about their health care (participant 3). Regarding the changing spectrum of germs and viruses and the resulting need for AI-enabled systems, GP 1 referred to the outbreak of the COVID-19 pandemic.

##### Stakeholder Influences

Another environmental influence was *stakeholder influences*, which indicated how certain groups of people and organizations influence GPs’ opinions. The interviews revealed that patients and institutions are key stakeholders in this context. GP 7 said the following:

I think we can be influenced [by the patients’ opinions] because, in the end, a medical practice follows the market like a small business. If the patients want [AI technologies] and demand [AI technologies], more and more practices will offer it.

Participant 7

However, the GPs also stated that they do not expect patients to disapprove of AI-enabled systems (participant 11). In contrast to the patients’ opinions, the GPs agreed that the opinions of institutions such as the German Society of General Medicine (Deutsche Gesellschaft für Allgemeinmedizin und Familienmedizin) or the German General Practitioners Association (Deutscher Hausärzteverband) have key roles in the formation of German GPs’ attitudes. GPs place trust in these institutions and regard them as scientific and validated committees of their profession (participant 11). Supported by the fact that physicians wish to receive more recommendations on which technologies they should use in practice, the influence of these institutions’ attitudes is evident (participant 7).

##### Media

The concept of *media* referred to all informative sources in which physicians had heard or read about AI-enabled systems. As most participants had not yet worked with AI-enabled systems, we assume that the media strongly contributes to AI literacy, which describes what GPs believe AI is and can do. A participant said the following:

Except for what I have read about it in medical journals, [I hardly come in contact with AI].

Participant 11

GP 14 suggested that physicians should be informed about AI technology via regular journal articles.

##### Information Technology Infrastructure

Another factor that influenced attitudes was the often-inadequate *information technology infrastructure* in physicians’ practices. In the event of technical problems, AI-enabled systems cannot be used properly or at all, which can undermine optimal patient care. Physicians are skeptical about AI-enabled systems in this regard and prefer the established ways of performing their routines, as they cannot rely on the overall infrastructure, which needs integration of AI technologies to function properly. In this context, a participant mentioned the following:

If my system goes down, my AI is on standby, then sorry, I can’t diagnose, my system strikes out. That is why it’s nice to be able to write down with a pen on paper what a patient has and has received.

Participant 16

#### Individual Characteristics

*Environmental influences* are external influences, whereas *individual characteristics* are determinants that describe a physician as a person and include character traits, demographic specifics, and knowledge. Although there are many individual characteristics, we found that age and affinity with technology are particularly relevant to the GPs.

##### Age

The participants who mentioned *age* disagreed on whether it has a role in determining their attitudes. GP 10, an older physician, said the following:

I am convinced it needs much work because there is certainly much resistance, which clearly depends on age.

Participant 10

Whereas GP 11, a younger physician, stated the following:

I also know young colleagues who are my age, and they also have strong reservations [regarding AI].

Participant 11

Thus, we included *age* as a relevant characteristic and leave future research endeavors to challenge its influence on a larger scale.

##### Affinity With Technology

Another influencing factor was *affinity with technology*, which indicated whether being open to new technologies supports a positive attitude toward AI-enabled systems. A participant said the following:

Well, there are also people in my generation who were already technically inclined [...]. So, I think that’s the key to why people [would use AI] or not.

Participant 18

#### Minimum Requirements of AI-Enabled Systems

##### Overview

Besides the above-mentioned categories and concepts, the interviews also revealed *the minimum requirements of AI-enabled systems*, which are preconditions that must be met for GPs to contemplate using AI-enabled systems. Although many of the requirements were thematically related to *expectations* and *concerns*, our qualitative data collection allowed us to distinguish between the attitude determinants and the essential and must-have criteria. We will now explain the 6 identified minimum requirements and underline their intensities with statements from the interviews.

##### Time Efficiency

Most participant statements that expressed demands of AI-enabled systems contributed to the minimum requirement of *time efficiency.* GPs need AI-enabled systems to be fast and easy to use, as they have limited time for each patient consultation. A participant mentioned the following:

First of all, [AI] should be fast. There is always time pressure.

Participant 14

Also, participants stated that AI-enabled systems must not take additional time, as this would keep a physician from performing essential tasks (participant 15). Thus, the focus was also on practical relevance and system compatibility with existing practice ISs. The participants demanded a self-explanatory design that can be operated quickly and in a few simple steps. The time component’s importance in the use of AI-enabled systems was shown by GP 15, who had already tested an AI-enabled system and decided against further use stated as follows:

[...] [the use of AI] took me far too long

Participant 15

##### Diagnostic Quality

Besides the time components, *diagnostic quality* was mentioned as another key requirement of AI-enabled systems. For physicians to consider the use of AI-enabled systems, the AI-enabled system must be validated, must not make mistakes, and must provide accurate diagnoses so that there is no threat to patient care (participant 7). Furthermore, some participants demanded accurate diagnoses and even better results through AI-enabled systems compared with human engagement, because otherwise, AI-enabled systems would be obsolete (participant 2). In addition, AI-enabled systems must be evidence-based and must follow guidelines. In this context, GP 10 said the following:

[AI must be] scientifically grounded and must provide validated results that [the physician] may not be able to produce in their entirety.

Participant 10

##### Data Security

Participants also named guaranteed *data security* as a requirement for using AI-enabled systems. The physicians justified this requirement with concerns about privacy and misuse of data and they do not want patient and physician data to be accessible to anyone. A participant explained as follows:

Of course, it is also important to me that there is corresponding data security. I do not want the patients and us to be completely transparent. That is certainly not in the overall interest.

Participant 10

Data security issues were the second reason along with time expenditure that made GP 15 decide to refrain from further using that AI-enabled system.

##### Economic Viability

*Economic viability* summarized the statements regarding AI-enabled systems’ affordability and questions about financing them. In this regard, GP 2 mentioned the following:

If they are affordable [then I would use AI applications].

Participant 2

Furthermore, the participants expressed their willingness to use AI-enabled systems based on how the technology is financed and stated that the cost–benefit ratio must be consistent.

##### Transparency

*Transparency* and thus the comprehensibility of AI algorithms is another key requirement of AI-enabled systems. To trust AI-enabled systems, it was important to the GPs that the proposals submitted by the AI-enabled system are comprehensible. Thus, a participant said the following:

I must know how [AI] obtains information and how [it] works.

Participant 11

##### Autonomy

*Autonomy* represented another requirement, indicating that an AI-enabled system must be self-managed by the providers. Using the technology is feasible only if a physician can continue to work autonomously and the next treatment steps are not decided by an AI-enabled system. However, the participants had a negative attitude toward intervention in a physician’s self-determined work. A participant explained as follows:

I would participate only [on a] voluntarily [basis].

Participant 15

## Discussion

### Principal Findings

**Figure 2:**
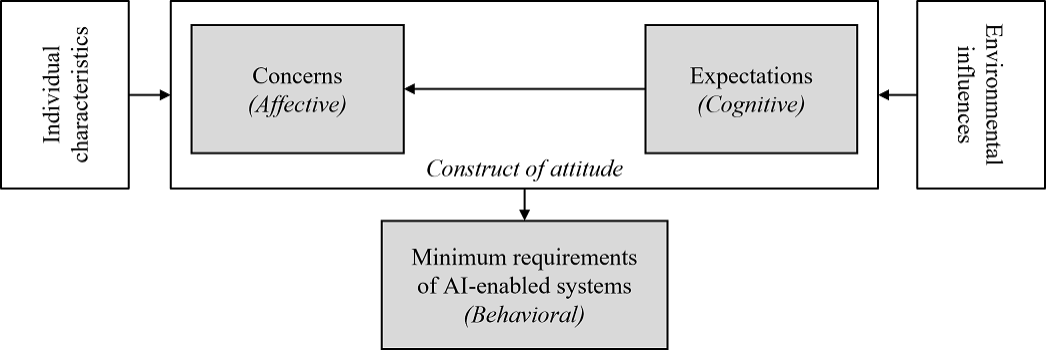
Model of the general practitioners’ determinants of attitudes toward artificial intelligence (AI)–enabled systems.

We now discuss GPs’ *attitude determinants* regarding AI-enabled systems in GP care and the relationships among these determinants. We conflate our findings to propose a model (Figure 2) and derive theoretical and practical contributions. Considering the lack of existing solutions and experiences of GPs with AI-enabled systems, our findings emphasize the relevance of GPs’ *AI literacy*. Hence, the interview statements and the resulting discussion are based on the GPs’ knowledge of AI, whether this is factual. The results underline that the participating physicians formed an opinion, even if they, as potential end users, did not have the necessary knowledge to understand the technology comprehensively or differentiate AI-enabled systems from knowledge-based CDSSs. Given that this will be the case for a large proportion of solely medically educated GPs, it is more important to investigate the determinants of attitude in rich detail. In doing so, research and practice can derive levers for the successful adoption of AI-enabled systems. Thus, and as the verisimilitude of GPs’ AI literacy is debatable, it emphasizes and gives important clues to understanding their attitudes and implications for practice.

Our attitude determinants *concerns*, *expectations*, and *minimum requirements of AI-enabled systems* corroborate the 3-component model of attitude proposed by Rosenberg et al [41]. The data analysis revealed the following: (1) the identified *concerns,* which represent the participants’ expressed emotions toward AI-enabled systems and refer to the affective component of attitude; (2) the identified *expectations,* which picture GPs’ beliefs toward AI-enabled systems and address the cognitive component; and (3) the identified *minimum requirements of AI-enabled systems,* which are preconditions that must be met for GPs to contemplate using AI-enabled systems and address the behavioral component of attitude.

However, as the relationships among these 3 determinants lack consistency and dependency according to our findings, we could not confirm the 3-component model [43]. Instead, the interviews revealed that GPs’ *concerns* and *expectations* form the *minimum requirements of AI-enabled systems*. This approach is consistent with the 2-component model of attitude, which indicates that the affective and cognitive components explain the behavioral intention [44]. For instance, the participants clarified that concerns about data misuse trigger the GPs’ demand for data security in AI-enabled systems. The importance of data security in health information technologies is not a novelty but rather a recurring theme in practice and research [71,72]. Another example is the expected time expenditure when using AI-enabled systems, which leads to the requirement that AI-enabled systems must be time efficient and simple to use. As for most health complaints, GP care is the first point of contact and GPs must treat a large number of patients. For instance, in Germany, GP care is one of the most frequently used health care services, with >200 consultations per week per physician [73] and an average physician–patient contact time of 7.6 minutes [26]. Thus, GPs are always under time pressure, which is why every additional action or additional use of new technologies must be well considered [74]. In part, these constraints in GP care are owing to an aging population [74]. GP consultations increase as age correlates with physician visits, particularly in primary care, where a high service use level by the older population is significant [75]. Besides older patients, the aging population also causes an increasing GP shortage owing to retirements and insufficient numbers of successors to GP care [76]. These interdependent developments further reduce the time available for a GP to make an initial diagnosis, which decides whether a patient receives the correct follow-up treatment, is treated at the right time, or receives treatment at all. Thus, a GP’s decision strongly impacts the course of treatment and outcome quality [28]. Consequently, increased workload and diagnostic suggestions with the potential to harm patients resulting from the use of AI-enabled systems would likely hamper technology adoption by GPs [77]. On the basis of our findings, we assume that GPs would not use AI-enabled systems if these require additional time or harm patients, despite their benefits. Thus, we consider *diagnostic quality* and *time efficiency* to be the most important *minimum requirements of AI-enabled systems*. Obligations to use AI-enabled systems by regulations or by superiors are neglected in this assumption.

However, we found not only minimum requirements of AI-enabled systems to be influenced by concerns and expectations but also *concerns* and *expectations* to be interrelated and form a construct of attitude. For instance, the participants’ concern of being replaceable was caused by their perception of AI-enabled systems formulating more accurate diagnoses than physicians. However, most of our participants did not fear being replaceable, as AI-enabled systems are unable to have and perform human competencies such as empathy and clinical reasoning. Similar conclusions were made by Oh et al [78] who conducted a web-based survey with physicians with the result that most of the participants do not believe that AI will replace physicians. In GP care, decisions are often made with incomplete and fragmented patient-specific information, requiring human competencies such as experience, intuition, and clinical reasoning [79]. Furthermore, in GP care, human competencies are of particular importance to develop a physician–patient relationship. To gather relevant information for the decision-making process, GP care places great importance on interpersonal continuity in the physician–patient relationship [39]. Especially regarding GPs’ gatekeeping role and their focus on an emotional bond in medical service provision, this interpersonal relationship is valuable because it enables the therapeutic benefit of improved continuity of care and more holistic and individualized treatments [32]. In summary, interpersonal interaction with patients is very important to GPs, whereas the GPs assume AI-enabled systems to have an insufficient ability to recognize and incorporate important individual aspects gained through the interpersonal relationship. Thus, where and when AI-enabled systems in GP care are useful is to be critically reflected [80]. Considering the potential of AI-enabled systems and their limitations reported in other research streams, we consider hybrid human–AI decision-making a promising scenario to mitigate the weaknesses of each other [36]. Enabling this scenario requires a profound understanding of GPs’ barriers to adoption [80], underlining the relevance of our identified attitude determinants.

We also found *individual characteristics* and *environmental influences* to determine GP’s attitude toward AI-enabled systems*.* Regarding individual characteristics, our results for the influence of GP’s age are inconclusive. The GPs in our sample presumed that both old and young physicians would have a negative attitude toward AI-enabled systems. However, both old and young participants in our sample generally had a positive attitude toward AI-enabled systems. As this may be owing to a bias in our sampling, we encourage further examinations of age as an attitude determining *individual characteristics.* Regarding *environmental influences*, our respondents indicated that a positive attitude from institutions such as the German General Practitioners Association would positively impact their attitudes toward AI-enabled systems. Moreover, GPs’ individual context such as office size and facilities (ie, *information technology infrastructure*) might prove themselves in further studies as determinants for GPs’ attitude toward AI-enabled systems. By uncovering *individual characteristics* and *environmental influences* as attitude determinants, we found similarities to the factors *social influence* and *age* of the UTAUT. Albeit, in the UTAUT, these determinants influence the intention to use [48,53,54]. However, in contrast to our findings regarding environmental influences, Jeng and Tzeng [81] concluded that social influence does not affect physicians in Taiwan in adopting CDSSs. This divergence may stem from different cultures, differences in medical education and practice, AI characteristics, and GPs’ AI literacy, compared with more established CDSSs. We leave it to future research to further explore these relationships regarding environmental influences.

Furthermore, our findings explicate that the consideration of the affective component of attitude is crucial in the medical context despite being neglected often in well-known theories of behavior and acceptance research [54,82,83]. Our interview data show that GPs’ concerns about data privacy and patient safety have high importance in the context of patient care and must not be endangered. AI-enabled systems can mitigate cognitive errors resulting from, among others, GPs’ fatigue or distraction [23]. Thus, diagnostic accuracy and patient safety increase [84]. However, at the same time, the integration of AI technologies can also lead to biases such as automation bias [85]. By blindly relying on the AI-enabled systems’ suggestions, physicians would no longer critically review them, which can reduce accuracy [86] and increase medical errors [87]. Whether AI-enabled systems promote or minimize cognitive biases depends on how they are used [84]. As AI-enabled systems bear certain concerns, such as the fear of being negatively biased by AI-enabled systems’ suggestions, the affective component of attitude also plays a key role in the context of AI. Eventually, the affective component is particularly relevant when investigating GPs’ attitudes toward AI-enabled systems. Detecting concerns in the early stage can positively determine GPs’ attitudes. When GP care comes into widespread contact with AI technologies, this form of attitude can contribute to a positive intention to use, which in turn lays the foundation for successful implementation.

Besides theoretical contributions, we derived valuable implications for practice by reflecting on GPs’ attitudes before the use of AI-enabled systems and familiarization with the technology. We suggest making the topic of AI more prominent in politics, health-related associations, and stakeholder institutions of GP care. Via these institutions, knowledge and education on AI-enabled systems can be offered, thus improving GPs’ AI literacy. This allows for the mitigation of concerns such as the *change of the physician–patient relationship* and thus, the diminution of restraints is possible. For this purpose, the distribution of evidence-based information via GP-specific journals and the involvement of advocacy groups are highly recommended, as the GPs value their viewpoints. However, it is also important that potential users are not only informed about the potential of AI technology but also about its limitations and shortcomings on the basis of evidence. In this way, physicians can be empowered to use AI-enabled systems in a reflective manner and thus, for example, prevent automation bias.

Moreover, the identified *minimum requirements of AI-enabled systems* are of particular interest concerning the practical implications. First, AI-enabled systems must be programmed and designed to make its use as easy and fast as possible as stated by participants and widely spread in the literature on user-centricity [88]. Second, AI-enabled systems must be reliable and free of errors to prevent any harm to patients. In addition, AI-enabled systems must ensure data protection and allow the GP to work autonomously. In addition, politics and health insurance companies should consider monetary subventions for AI-based systems because a remarkable result of the review by Ajami and Bagheri-Tadi [89] is the positive influence of financial support on physicians’ willingness to use and engage with technologies [89].

Furthermore, AI-enabled systems may foster so-called *black-box-medicine*, as decisions are less transparent to the patient and to the GP. With this lack of transparency, various types of biases may occur, both for the end users and the AI-enabled system. Such biases may result in patient security, data, and privacy concerns [84,90]. Therefore, along with the responsibility of making an AI-augmented diagnosis, there is also the need to create accountable structures for patient-related outcomes. In a recent study by Khullar et al [91], physicians believed that vendors or the employing health care organizations should be held accountable for AI-induced errors, whereas the general public believed that the physicians themselves should be liable. We see suitable liability regulations and their implications for GP’s attitude determinants as a promising field for further research.

Furthermore, AI-enabled systems should be developed to diagnose rare cases because GPs assume that they are faster in routine cases than using AI technology. This information can help developers to narrow the application area and to create better-fitting software solutions. This result also indicates that integrating AI technology is not the solution for every problem. Rather, a critical assessment must be made regarding when using an AI-enabled system makes sense and improves decision-making and when this is not the case. Especially when it comes to human competencies and interpersonal relationships, AI-enabled systems cannot replace GPs. Rather, AI-enabled systems should be designed to free up GPs’ time so that they have more time to nurture relationships with their patients, which is of particular relevance for diagnosis in GP care. Our findings may serve for a better understanding of how to design AI-enabled systems in a conducive manner and how to foster GP’s acceptance in the later adoption of such systems.

### Limitations and Future Research

Although we rigorously followed our designed research approach, our study has limitations, some of which are bound to the choice of a qualitative-explorative approach. By design, qualitative interviews do not focus on drawing conclusions for entire populations, which affects the generalizability of the results. Nevertheless, a qualitative approach is appropriate before a quantitative study when dealing with a new and emotionally charged topic. This approach is reinforced by recent research that puts traditional IS adoption models to the test for AI; thus, calling for in-depth reflections [21]. Blease et al [39] also recommended a qualitative approach, as they reported lack of detailed information on GPs’ views of AI-enabled systems owing to their quantitative approach. Furthermore, conducting interviews just in 1 country, more precisely, in 1 geographic area within that country might be a limitation of our study. As depicted in existing research, attitudes toward technology might differ between people living in rural areas and urban areas [92]. We recommend collecting data in other countries and conducting cross-country studies to detect differences among these settings. Furthermore, GPs’ mostly basic AI literacy is another limitation of our study. Although all study participants were given the same definition of AI-enabled systems at the start of the interview, their statements reflect different understandings. However, the early consideration of the GPs’ attitudes, regardless of their technical knowledge is important to identify barriers to implementation at an early stage and derive basic conclusions for AI system design. We must also assume that only GPs who are interested in AI-enabled systems might have a general affinity with technology or who have a strong opinion on AI agreed to be interviewed. This could also explain why none of the participants had a solely negative attitude toward AI-enabled systems.

We further suggest examining the role of the affective attitude component, because we revealed the importance of the identified concerns in our study; whereas, in well-known theories of technology acceptance, this component is often neglected. A closer examination of the affective component will make it possible to determine the extent to which it is relevant in the medical and IS contexts.

### Conclusions

AI-enabled systems are considered as promising solutions to enhance both the effectiveness and quality of health care. Especially in GP care, which is the first point of contact for most medical needs, physicians deal with a shrinking physician–patient time and incomplete or sometimes incorrect information. Here, AI technology promises new solutions to support physicians and decrease diagnostic errors that lead to extensive consequences. Although the application potential of AI-enabled systems in health care has been widely discussed theoretically and conceptually, a widespread application in the professional practice of GPs is still dreams of the future. To tap the undisputed potential of AI-enabled systems in practical use, a fundamental investigation of the technical systems and social actors is required. As academic research, in this respect, is still in its infancy, we investigated the attitudes of GPs toward AI-enabled systems. Thereby, we seek to contribute to a better understanding of GPs’ attitudes, which is crucial for developing and implementing suitable AI-enabled systems. Thus, we used in-depth qualitative-explorative interview data with German GPs and proposed a preliminary research model. We identified three determinants of GPs’ attitudes: *concerns*, *expectations*, and *minimum requirements of AI-enabled systems*. Furthermore, we revealed *individual characteristics* and *environmental influences* as the 2 conditional determinants of GPs’ attitudes toward AI-enabled systems. The findings emphasize the importance of attitude’s affective component at the interface of medical and AI research. Moreover, the findings show that diagnostic quality and time efficiency are mandatory for GPs to even consider the use of AI-enabled systems. Therefore, integrating user groups’ attitudes and needs is a fundamental prerequisite for user-centered design, which leads to a higher willingness and inclusion of the systems into everyday use. Considering that the GPs in our interview study predominantly corroborated AI-enabled systems’ seminal role in the future of GP care, our findings may serve as a foundation for future research. Besides investigating the attitudes of user groups in other fields in the health care system, research endeavors should also focus on how the attitudes of GPs toward AI-enabled systems can be proactively promoted. In addition, future work should include and conflate findings from related research areas such as human-computer interaction, psychology, sociology, and computer science to account for AI’s interdisciplinary implications for health care.

## Appendix

Multimedia Appendix 1 Interview guideline.

## Abbreviations

AI: artificial intelligence
CDSS: clinical decision support system
DSS: decision support system
GP: general practitioner
GTM: grounded theory methodology
IS: information system
UTAUT: unified theory of acceptance and use of technology
