# Supplementary material for: General Practitioners’ Attitudes Toward Artificial Intelligence–Enabled Systems: Interview Study
Source: J Med Internet Res. 2022 Jan 27;24(1):e28916. doi: 10.2196/28916 (PMC8832268; doi:10.2196/28916)
Supplement: Multimedia Appendix 1 [file jmir_v24i1e28916_app1.docx]

Appendix 1

Note: This interview guideline has been translated from German into English.

**Introductory questions**

1. Would you be so kind as to briefly introduce yourself and your activities working as a GP?
2. What are the greatest challenges confronting you in your work as a GP?
3. To what extent do you already use systems/ tools which support you in diagnosis?
4. For which treatment cases do you consider diagnostic support systems useful?
5. Where do you see the greatest added value in the use of diagnostic support systems?
6. Is there a need for more support systems for diagnosis on the market? And if yes, how should these systems look like?

**Key questions**

In the following, we would like to talk to you about AI-technologies in diagnosis.

1. What is your understanding of AI?

*In the context of our research project, we understand AI as a human-programmed computer system that uses self-learning algorithms to provide decision support, such as diagnostic suggestions. For example: The physician types all the data of a patient (e.g., blood values, findings from medical history, diagnostic results, etc.) into an AI-based solution, which recognizes patterns in the data and provides diagnostic suggestions.*

1. Following this understanding of AI: Which thoughts come to your mind when you think of AI?
2. How do you feel when you think about integrating AI into your daily work?
3. Do you have more of a positive or negative feeling when you think about this idea?
4. Would you use the AI to get diagnostic suggestions even before you have expressed your own thoughts, or rather to test the diagnosis you have made?
5. So far, how much experience have you had with AI in medicine?
   1. (In case of no experience)
      In your opinion, what are the reasons why you have never been exposed to AI in medicine?
   2. To what extent do you perceive the familiarization of general practitioners with AI is important?
6. Please share your opinion: To what extent will artificial intelligence play a role in family medical care in the coming years?
   1. (In case of no importance assigned to the topic)
      Could you please tell me in more detail why you think so?
   2. How do you think an AI could help GPs in the diagnostic process?
   3. How exactly would the use of AI affect your daily work and patient care?
7. How do you perceive the diagnostic sentiment towards AI in your working environment?
   1. Who would most likely influence your attitude towards AI?
   2. To what extent would your attitude be influenced?
8. When would you decide for or against the use of AI-based technologies in diagnosis?
9. Which chances and which risks do you associate with the use of AI in diagnosis?
   1. What do you consider as the greatest added value in the application of AI technologies?
   2. What are your biggest concerns when you think about using AI?

**Closing question**

1. Before we finish the interview, would you like to add anything we have not discussed yet?
